# HUPA: A corpus of disordered and normophonic voices in Castilian Spanish

**DOI:** 10.1016/j.dib.2026.112676

**Published:** 2026-03-16

**Authors:** Juan C. Puerta-Acevedo, Maria F. Alcalá-Durand, Janaína Mendes-Laureano, Julián D. Arias-Londoño, Juan I. Godino-Llorente

**Affiliations:** Escuela Técnica Superior de Ingenieros de Telecomunicación, Universidad Politécnica de Madrid, 28040, Madrid, Spain

**Keywords:** Acoustic analysis, Castilian Spanish, GRBAS scale, HUPA dataset, Pathological voice detection, Voice disorders dataset

## Abstract

Advances in the evaluation of voice quality and the automatic screening of voice disorders are currently constrained by the scarcity of large, publicly available datasets that include speakers of diverse native languages, which hampers the reproducibility and further development of research in this field. To mitigate this limitation, the present manuscript introduces the HUPA corpus, a comprehensive clinical dataset comprising sustained-vowel voice recordings from 440 native Castilian Spanish participants, including 239 healthy controls and 201 individuals diagnosed with a wide range of voice disorders. In addition to the audio recordings, the corpus provides subjective evaluations of voice quality performed by three expert raters using the GRBAS scale (Grade, Roughness, Breathiness, Asthenia, Strain), as well as a set of acoustic parameters that are automatically extracted from each voice record to offer an objective characterisation of vocal attributes. To the best of our knowledge, the HUPA corpus is the largest open-access, clinically validated voice-disorders database available of Castilian Spanish speakers. The HUPA corpus, therefore, constitutes a substantial resource for the investigation of the acoustic manifestations of voice disorders in Castilian Spanish participants and for the development and benchmarking of automatic voice pathology assessment methods. It has already supported multiple studies on automatic screening and acoustic feature design, illustrating its potential as a reference resource, while the broader challenge of developing robust, cross-corpus screening systems that generalise across languages, recording conditions and clinical populations remains an open research question.

Specifications Table

**Table:** 

Subject	Health Sciences, Medical Sciences & Pharmacology
Specific subject area	Automatic Voice Condition Analysis; Otorhinolaryngology; Biomedical Signal Processing.
Type of data	Table (Excel metadata), Audio (WAV files), Code (MATLAB/Python scripts).
Data collection	Instruments: Kay Elemetrics CSL 4300B system; Shure SM48 cardioid dynamic microphone. Software: AVCA-ByO toolbox (acoustic analysis). Conditions: Acoustically treated room (reverberation < 0.25 s, noise < 40 dB SPL). Microphone at 15 cm, 45 degrees azimuth.
Data source location	Institution: Hospital Universitario Príncipe de Asturias and Universidad Politécnica de Madrid. City/Country: Madrid, Spain.
Data accessibility	Repository name: Zenodo. Direct URL to data: https://zenodo.org/records/17704572 Instructions for accessing these data: Although the corpus is not a fully anonymous download, the repository and its documentation are openly accessible, and data are routinely shared for non-commercial research under a standard Data Use Agreement. Prospective users should copy the full text of the use agreement into the ‘request message’ field in the Zenodo repository, together with their institutional and contact details. Requests that explicitly agree to the terms and conditions will be evaluated for access to the corpus.
Related research article	Feature Extraction and Acoustics: [[1], [2], [3], [4], [5], [6], [7]] Deep Learning and Advanced Modelling: [8,[9], [10], [11]] Cross-Corpus Validation and Robustness: [[12], [13], [14], [15], [16], [17], [18]]

## 1 Value of the Data

• This dataset provides a comprehensive resource for the analysis of voice disorders of **Castilian Spanish speakers**, filling a gap in publicly available corpora for this language.
• It includes a balanced cohort designed for screening purposes (**239 healthy controls and 201 pathological patients)**, reducing the imbalance commonly observed in other voice disorders datasets. The sex distribution within each group reflects the epidemiological balance commonly reported in literature (∼40 % men and ∼60 % women).
• The data contains expert perceptual evaluations using the **GRBAS scale (Grade, Roughness, Breathiness, Asthenia, Strain)** and a set of acoustic parameters that are automatically extracted from each recording, enabling the development and benchmarking of automatic screening systems against clinical gold standards.
• Recordings were acquired under **strictly controlled clinical conditions** (fixed protocol, acoustically treated room, professional equipment), ensuring technical homogeneity for reliable signal processing research.
• The corpus supports cross-database benchmarking and can be used not only to develop **robust, native language and corpus-independent machine-learning models** for pathological voice detection, but also to explore and validate acoustic biomarkers that remain stable across corpora, recording conditions, and clinical populations.

## 2 Background

Traditional clinical assessment of voice disorders combines perceptual-auditory scales (e.g., GRBAS and CAPE-V) [19] with objective acoustic features extracted from the acoustic trace. This combination provides more complete clinical evidence than a single modality and can support the identification of specific disorder types. Although perceptual ratings remain fundamental for diagnosis, they are inherently subjective, time-consuming, and dependent on the clinician’s expertise. Consequently, the development of Automatic Voice Condition Analysis (AVCA) systems is receiving increasing attention, as they provide objective, reproducible, and non-invasive means to support clinical diagnosis and long-term monitoring of voice disorders [5,20]. In practice, many AVCA studies focus on binary screening, whereas multi-class modelling of specific disorder subtypes is less common and typically more challenging —highlighting the need for larger, well-annotated clinical corpora with reliable labelling. Therefore, robust AVCA relies on high-quality corpora of pathological and normophonic (i.e., healthy control) voices recorded under controlled conditions. Since each corpus represents only a subset of a much broader population, a careful design of the datasets is required to minimise the bias related to recording conditions, age and sex distributions, and population demographics.

Unfortunately, to date, the number of open-access corpora of voice disorders is quite limited, and there are no widely accepted standards for creating such databases, significantly hindering advances in the field. Recent evidence confirms that, despite the growing interest in the field, the research concentrates on a small set of widely reused corpora. For instance, a comprehensive review covering the period 2015–2024 identified approximately 35 distinct voice disorders databases in published studies. Besides, most of these studies relied on a single corpus, and experiments were disproportionately concentrated on a handful of well-known resources, notably the Saarbrücken Voice Database (SVD) and the Massachusetts Eye and Ear Infirmary (MEEI) voice disorders database, with others —such as HUPA (Hospital Universitario Príncipe de Asturias), VOICED (VOice ICar fEDerico II Database), and AVPD (Arabic Voice Pathology Database)— used less frequently. In contrast, many of the remaining databases are of privative use, not being available for the research community (and often reported and/or evaluated in one single research publication), which limits reproducibility of results, cross-corpus generalisation and independent benchmarking [21]. This lack of standardisation was highlighted decades ago by Fourcin [22], who argued that objective comparisons across research centres, participants, and languages require harmonised criteria, covering: **(i)** the recording protocol and technical conditions (e.g., microphone type/placement, environment, sampling rate and calibration); **(ii)** elicitation material and participant instructions; **(iii)** clinical labelling and metadata (e.g., diagnosis, severity ratings, demographics); and, **(iv)** transparent and comparable analysis procedures. Fourcin also emphasised that, beyond technical standardisation, an essential characteristic of any corpus intended for research is its public availability with sufficient documentation, enabling objective benchmarking and reproducibility.

Building on these criteria, the practical utility of a voice disorders database depends on how consistently recording protocols are documented (e.g., device/channel, environment, calibration…), how speech tasks are specified and standardised, and how annotation and metadata (e.g., diagnosis, severity, perceptual evaluations, demographics, and relevant clinical context) are captured. This protocol and annotation-oriented view is consistent with methodological guidance in health-related speech research and the broader literature on voice data collection and annotation practices [[23], [24], [25]].

Up to date, the number of resources available to the scientific community to develop robust AVCA systems is still limited, and standardisation remains lacking. This situation persists despite major technological advances since the early 1990s: high-quality digital audio can now be recorded with inexpensive and ubiquitous devices (e.g., laptops, tablets, and smartphones), open software is available for recording, storage means are affordable, and powerful cloud-based data management platforms are available. Despite of this, progress has often relied on privative corpora developed by individual research groups to train, test, and validate their own systems, taking little advantage of the technical advances, and often reporting results that vary substantially when the same system is trained and tested on different corpora [26] due to significant differences among them (e.g., native language, specificities of the cohorts, inclusion criteria, microphone and recording-device specifications, room acoustics, background noise, etc.). This variability complicates direct comparisons between methods [27], making the results difficult to extrapolate, and also limiting their potential translation to the clinic. Therefore, the number of available open-access corpora is essential for cross-corpus benchmarking and for developing robust corpus-independent AVCA systems.

In this context, we will refer to the Findable, Accessible, Interoperable, and Reusable (FAIR) principles [28] which provide widely adopted guides to ensure that a dataset can be discovered, accessed under clearly defined conditions, integrated with other resources, and reused reliably —thereby supporting reproducibility and cross-corpus benchmarking. HUPA comes to partially fill the aforementioned needs by addressing the FAIR principles and Fourcin’s insights.

### 2.1 Voice disorders databases of privative use

In this section, we address the most relevant datasets that are described in the literature but not openly released to be used by the scientific community, or with no clear access mechanisms reported (no download link and/or no stated request or agreement process).

Most existing voice disorders databases are of privative use, lacking distribution mechanisms, or being only available under restrictive conditions. A recent survey on voice data practices revealed that most voice disorders databases are of privative use and have never been shared with the research community, and that only 28 % of centres utilise standardised acquisition protocols [29].

Several factors contribute to the limited public release of voice disorders datasets, including the possibility to de-identify the patients, data privacy constraints, intellectual-property rights associated with the corpus, and the burden associated to long-term maintenance of the data [30]. These barriers are consistent with those evidenced for health data sharing in a broader sense, where privacy, governance, legal/ethical aspects, and resource constraints are recurrent obstacles to share sensitive health data [31]. When sharing is feasible, the procedure includes a rigorous de-identification of the patients, metadata minimisation, pseudonymisation of potential identifiers, the use of controlled elicitation tasks (e.g., sustained vowels or read speech), and tiered-access models (e.g., data-use agreements and secure access) [[32], [33], [34], [35],^36^].

At the large-scale end, several corpora of voice disorders have been compiled through multi-centre clinical collaborations, yet they remain accessible only to their developers. For example, a multicentre corpus collected by the Belgian Study Group on Voice Disorders comprises more than one thousand normal and pathological voice recordings [37]. In France, the Laboratoire Parole et Langage has recorded and curated big corpora, including the MTO dysphonia corpus —1530 dysphonic patients and 1953 recording sessions comprising sustained vowel tasks, read text and singing, with GRBAS ratings available for 1766 sessions— and the AHN dysarthria corpus —990 patients and 160 controls, with audio and aerodynamic recordings, and extensive clinical metadata—. Despite their scope and clinical utility, these resources are of privative use [38].

Alongside these flagship efforts, the literature reports many other corpora of privative use. The MAPACI database, for example, comprises 48 sustained vowel task recordings from 24 vocally healthy controls and 24 patients with voice disorders [17] specifically shared for a telemonitoring work, it is not available through a public repository [39]. Resources with restricted-access have also been reported for specific populations, longitudinal recordings of male patients with and without laryngeal cancer [40].

Beyond the aforementioned resources, additional corpora of privative use have been collected by individual centres to evaluate different signal processing or modelling strategies. Recent systematic reviews of AI-based voice disorders detection also mention this practice [17]. The studies in [[41], [42], [43], [44], [45], [46], [47], [48], [49], [50], [51], [52], [53], [54], [55], [56], [57], [58]] provide examples of these corpora. The recording tasks vary across studies. Some corpora record sustained vowels (e.g., /ae/) [42,53]; and others use read speech [41] or connected speech [42,58].

A smaller but important subset of datasets of privative use go beyond sustained vowel to include connected or semi-connected speech [59] (e.g., isolated words, read text, and/or spontaneous conversations), which increases heterogeneity across protocols. Importantly, these resources also differ in the breadth of accompanying clinical metadata: some report perceptual severity grades/ratings during reading and conversation, whereas others provide only diagnostic stratification and audio. Examples include a clinical cohort combining perceptual assessments during reading, spontaneous conversations, and several recordings of the sustained vowel (/a/) [55]; multi-cohort Polish recordings including vowels, isolated words and read text [56]; French read speech balanced across perceptual grades [57]; a Dutch corpus with 661 stimuli and diagnosis counts [58]; and multilingual read-text resources reported with substantial recording volumes and specified storage formats [60,61]. Larger clinical cohorts with structured diagnostic grouping and standardised tasks have also been reported without open release [62].

UEX-Voice is another example of a corpus of privative use including benign vocal fold lesions. It was recorded in an ordinary clinical setting using a head-worn microphone. The corpus is based on the sustained vowel /a/ and includes 30 healthy controls speakers, 24 patients with nodules, and 30 patients with Reinke’s edema [14,59].

These corpora of privative use are not limited to Western European or North American speakers. For example, a Korean clinical voice cohort [63] has been reported with 1524 participants uttering the /a/ vowel, and reading a Korean passage. Besides, a Japanese speaking cohort [64] has also been reported with 336 voice recordings that combine sustained vowels and continuous speech.

To improve readability and comparability, Table S1 is provided in the Supplementary Material. It summarises the most representative examples, including cohort size, language, speech material, diagnostic stratification, demographics, recording conditions, and access modality. When key protocols or technical details are omitted in the associated publications (e.g., microphone type, recording setting, age range, or sex breakdown), these fields are marked as not reported (NR).

### 2.2 Open-access voice disorders datasets

The FAIR principles are widely used to guide data sharing and reuse. Public release alone does not guarantee full FAIR compliance, because FAIR also requires rich metadata, a clear data use license, and provenance. However, access can still be FAIR when it is controlled. The FAIR principles allow authentication and authorization when needed, as long as data are retrievable by their identifiers using a standard protocol. Thus, for better clarification of the access conditions, the following categories are used in this section: **(i) Open download** means that the data can be downloaded directly with no restriction at all; **(ii) Registration required** means that the data can be downloaded after prior registration or email confirmation, without signing a Data Use Agreement (DUA); **(iii) Derived data only** refers to corpora that release only parameters extracted from the acoustic waveforms, while raw audio waveforms are not released; **(iv) Controlled access** means public access, but with prior approval or a DUA.

Following these categories, the next paragraphs present the most relevant open-access voice disorders datasets.

#### 2.2.1 Open download

The Institute of Phonetics of Saarland University in Saarbrücken (Saarland, Germany) compiled and released the SVD corpus [65]. It collects recordings of 2043 participants recorded until 2004, of whom 687 are healthy controls and 1356 are patients with voice disorders. For each participant, audio and electroglottographic recordings of the sustained vowels /a/, /i/and /u/ were collected with normal, low, and high fundamental frequency levels, and also with a rising a falling fundamental frequency. The phrase ``*Guten Morgen, wie geht's Ihnen*'' (``Good morning, how are you?'') was also recorded. The duration of the sustained vowels is between 1 and 3 s. Among the pathological voices, there are examples of 71 different pathologies. The database was developed in collaboration with clinical partners, and while the main speech material is reported, publicly available documentation provides limited detail on the acoustic environment and the specifications of the microphone.

#### 2.2.2 Registration required

The Italian VOICED [66] contains 208 recordings of the sustained vowel /a/ (58 healthy controls and 150 patients with voice disorders) recorded under a standardised protocol. Although limited in size and class-imbalanced, it provides clearly documented metadata and includes explicit healthy controls, making it a valuable benchmark for pathological voice research.

The Perceptual Voice Qualities Database (PVQD) [67] contains 296 high-quality recordings of sustained vowels and sentences from adult speakers with and without dysphonia; 89 speakers reported no voice complaints, while the remaining recordings correspond to pathological voices of varying aetiologies and severity. The corpus is particularly suited for studies linking acoustic correlates to perceptual severity ratings.

#### 2.2.3 Controlled access

Crowdsourced initiatives have also contributed to the landscape of open-access resources, but access is not always by direct download. The UncommonVoice dataset [68] provides recording from 57 English speakers, 48 of whom have spasmodic dysphonia, and was recorded remotely with consumer devices. The speech material includes a sustained vowel, diadochokinetic sequences, sentences and short spontaneous descriptions, offering a complementary perspective under realistic home-recording conditions but is mainly limited to the study of spasmodic dysphonia.

The Advanced Voice Function Assessment Databases (AVFAD) [69] contains recordings from 709 European Portuguese speakers (363 healthy controls and 346 pathological patients) uttering sustained vowels, words, sentences and connected speech, accompanied by detailed clinical labels and a standardised evaluation protocol. Both datasets are obtained by request to the corresponding author.

Other publicly documented corpora are accessible under controlled conditions. The Far Eastern Memorial Hospital (FEMH) database (Taiwan; Mandarin) released a curated subset with a fixed train-test protocol [[70], [71], [72]]. The released subset includes the sustained vowel task (/a/) and recordings of read sentences. It was accessed only after approval for those participating in a challenge organized by the developers [[73], [74], [75]].

The Malaysian Voice Pathology Database (MVPD) was built around 382 participants (252 healthy controls and 130 dysphonic patients). The voice task is a 5 s sustained /a/ produced at comfortable loudness, and the database also includes clinical diagnosis and laryngostroboscopy data. It is available upon request to the corresponding author [17,76].

The AVPD [77] includes 366 speakers and records sustained vowels, running speech, and isolated words, with perceptual severity ratings. AVPD is also a well known database that is used by many studies, as highlighted in [21]. Access to AVPD is typically provided upon request to the corresponding author.

#### 2.2.4 Derived data only

Some datasets provide open-access but only share derived data extracted from the acoustic trace. Bridge2AI-Voice (v3.0) provides data derived from voice recordings, such as spectrogram-based representations and other features, from North American 833 participants, recruited from specialty clinics into five clinical cohorts (respiratory, voice, neurological, mood, and pediatric) and linked to detailed demographic metadata, clinical information, and validated questionnaires. Data were collected under a standardised protocol using a tablet-based application, with a headset microphone when possible, and include structured voice tasks such as sustained vowels (with some participants contributing with several sessions). The public release includes derived data only, not raw audio waveforms. The original audio recordings are available only through a controlled access companion release [78].

In the same way, [79], collects voices from speakers with vocal fold lesions, including Reinke’s oedema, but releases only acoustic descriptors and not the audio waveforms.

Overall, these open or conditionally accessible resources broaden the linguistic and clinical landscape of pathological voice corpora, but they remain fragmented in terms of access conditions, recording protocols and available metadata. Table S2 included in the supplementary material summarises the reported open-access and conditionally accessible voice disorders datasets, including their cohort size, speech material, participant grouping, recording conditions, available metadata, and access level, to provide a uniform overview beyond what can be included in the main text.

### 2.3 Commercial access

After open-access resources and privative use datasets, there is a third case that is important for historical reasons: commercially distributed corpora. These datasets are not open-access and they are not shared by request. However, they were widely used in earlier work, so they shaped benchmarking practice for many years.

A key example is the corpus compiled by the MEEI Voice and Speech Laboratory (Boston, Massachusetts, USA) [80]. It was distributed on CD-ROM since 1994 by Kay Elemetrics® (no longer commercially available). It contains recordings of 637 participants: 53 healthy controls and 584 patients with a wide range of voice disorders: organic, neurological, psychological, and traumatic. The material recorded includes the sustained vowel /æ/ and a reading of the Rainbow Passage. Notably, the sustained vowels recordings correspond to a stable mid-portion of phonation, excluding onset/offset, which limits analyses of vocal onset/offset behaviour. The entire corpus was recorded with a similar equipment but in two different acoustic environments and with three different sampling frequencies. This was the only database available for many years and therefore became, *de facto*, the standard corpus used for benchmarking purposes for a long time. Despite its obvious utility, this database has been criticised in the past for various reasons, including class imbalance, heterogeneous recording conditions (different environments and sampling rates), and the mid-portion vowel segmentation noted above [15].

### 2.4 Characteristics of existing corpora

Having reviewed both open-access and privative resources (with the latter discussed from 1998 onwards), we now summarise the recurring design choices and limitations that emerge across the literature. For clarity, these characteristics are grouped into six dimensions: **(i)** participant demographics; **(ii)** speech material and task design; **(iii)** pathology coverage and labelling strategies; **(iv)** recording protocol and technical conditions; **(v)** annotation, perceptual assessment and multimodal metadata; and, **(vi)** common limitations and synthetic extensions.

#### 2.4.1 Participant demographics

Across corpora, adult participants predominate, but sex and age distributions vary widely, often reflecting local clinical caseloads rather than balanced sampling. At a rough level, the reviewed corpora span multiple languages, with widely reused resources centred on English (e.g., MEEI, PVQD, UncommonVoice). In addition, several open-access corpora cover other languages, either by open download or under conditional access. These include German (SVD), Italian (VOICED), European Portuguese (AVFAD), Arabic (AVPD), Mandarin (FEMH) and Malaysian (MVPD). This heterogeneity is apparent in both centre-specific clinical resources and study-level datasets spanning multiple languages and settings [[45], [46], [47],^62^,^69^,^76^].

Where demographics are explicitly reported, sex ratios can be markedly skewed. For instance, VOICED (adults 18–70) is female-skewed in both the healthy group (37F/21 M) and the pathological group (98F/52 M), whereas several other corpora provide limited or no sex/age breakdowns. Some resources are explicitly restricted to particular demographic groups, such as female-only clinical cohorts [53,57] or male-only longitudinal recordings [40], which fragments demographic coverage and can limit representativeness.

#### 2.4.2 Speech material and task design

Although the recorded material ranges from isolated vowels to connected speech, there is a clear predominance of sustained phonation –especially /a/–, which offers a convenient and clinically feasible task and yields quasi-stationary signals for analysis. In addition, sustained vowels place minimal linguistic and cognitive demands, reduce articulatory/lexical variability, and better isolate phonatory function, enabling more comparable estimation of voice-quality markers across participants and centres [24]. Sustained vowels –often /a/ or set of vowels–, are central in many corpora of privative use [[43], [44], [45], [46], [47], [48], [49], [50], [51], [52], [53], [54]] and also remain prominent in open or conditionally accessible resources [[65], [66], [67], [68], [69], [70],[76], [77], [78], [79]]. Importantly, when this is reported, some corpora provide sustained vowels from onset to offset (e.g., AVPD), whereas others store only a stable mid-portion without onset/offset (e.g., MEEI and SVD), and others have inconsistent information about this (VOICED, PVQD, Uncommon Voice, and MVPD).

Many corpora and experimental studies also include connected or semi-connected speech (read text, phrases, words and sentences), motivated by their clinical relevance and by the desire to capture phenomena that may be attenuated in sustained phonation. Importantly, isolated words and read text provide a predefined linguistic target (i.e., the expected utterance), which facilitates direct comparisons among participants, as well as more controlled assessment of specific phonetic and articulatory attributes. Compared with spontaneous speech, these tasks reduce linguistic variability and support more standardised sampling across participants and centres. Corpora of privative use include French read speech with severity grades [57], Polish recordings with vowels, isolated words and read text [56], Dutch connected speech with diagnosis [58], and multilingual read speech datasets reported with large recording volumes and specified storage formats [60,61]. Open or conditionally accessible corpora likewise combine sustained vowels with words, sentences and connected speech, supporting complementary analyses across task types [[67], [68], [69],[75], [76], [77]]. Repeated recordings of the same task, often sustained vowels, are common. This can improve robustness and can also measure variation within the same participant [46,54,81].

#### 2.4.3 Pathology coverage and labelling strategies

Pathology coverage is often broad, with many corpora mixing organic, neurological and functional disorders, reflecting heterogeneous clinical referral patterns [45,47,62,69,76,82,83]. Other datasets focus on particular clinical entities or questions, such as laryngeal cancer [41], vocal-fold nodules [42,54], unilateral paralysis/paresis [46,53], or specific benign lesions in targeted clinical studies [14,59,79]. Some corpora employ coarse clinical groupings (e.g., healthy/diffuse/nodular) to support classification studies without fine-grained diagnostic resolution [48,49], whereas others report explicit diagnosis counts within pathological cohorts [43,46,47,50,58]. This diversity of clinical labelling complicates direct cross-corpus comparison and underscores the need for transparent reporting of diagnostic criteria.

#### 2.4.4 Recording protocol and technical conditions

Recording conditions range from acoustically treated rooms and professional equipment with controlled protocols to ordinary rooms and everyday devices, introducing substantial variability in noise, channel characteristics and microphone placement [54,68,69,76,77,82,83]. Technical parameters also vary markedly: studies report sampling rates from low-to-mid values (e.g., 11 kHz storage in a Lithuanian clinical corpus) [49] through 44.1 kHz and 50 kHz acquisition [53,61,65], with 16-bit quantisation commonly reported [45,49,50,54,58]. Such variability can inflate apparent performance differences across studies and makes it difficult to disentangle algorithmic gains from corpus-specific recording artefacts. Privacy and data security can affect the released data and how access is granted, especially for open or conditionally accessible corpora. Some datasets release raw audio with open download, registration, or controlled access. Others release only derived data to reduce de-identification risks. In Table S2 this information is included.

Another practical constraint is that many datasets contain short recordings. This can reduce statistical robustness and make results more sensitive to segmentation choices. Building large clinical corpora takes a long time and is limited by clinical workflow and disease prevalence. Longitudinal data can add more heterogeneity, because recording environments and equipment may change over time [40].

#### 2.4.5 Annotation, perceptual assessment and multimodal metadata

A subset of corpora provides rich multimodal information, which is valuable both for validation and for multimodal diagnostic systems. Beyond acoustics, some resources include physiological measures and structured clinical information, as exemplified by datasets underpinning clinical indices and/or pairing acoustics with physiological and endoscopic findings [82,83]. Detailed perceptual voice quality assessments are also present in some datasets, enabling explicit links between acoustics and perceived severity or voice quality; examples include graded connected speech [57], clinical cohorts with perceptual assessment alongside acoustics [55], and open corpora designed to study the link between acoustic measures and perceptual ratings [67,77]. Some corpora also report demographic data and survey information about voice use. This information can help clinical interpretation and can support models that distinguish disorder subtypes. However, many corpora do not report these data in a consistent way. Multimodal datasets combining acoustics with additional signals and clinical metadata have also been reported under conditional access, including resources associated with the FEMH line of work [[73], [74], [75]]. Nevertheless, rich annotation remains far from universal, and many clinically collected datasets are weakly documented from the perspective of reproducible signal processing.

#### 2.4.6 Common limitations and synthetic extensions

Across both open-access and privative corpora, recurring limitations include small sample sizes for specific disorders, short and heterogeneous recordings, demographic imbalance, inconsistent labelling schemes and variable documentation of recording protocols. These factors make benchmarking harder, reduce comparability between studies, and can hide whether performance differences come from better models or from corpus specific bias.

To mitigate data scarcity and class imbalance, the literature has explored synthetic and augmented resources for more than a decade. Early work focused on analytic synthesis of disordered vowels and controlled perturbations of sustained phonation [[84], [85], [86]]. More recent approaches use data driven methods to imitate dysphonic or dysarthric voices, converting healthy speech into pathological-like speech, and using neural vocoders to control the type and severity of the impairment. [[87], [88], [89], [90], [91]]. Although such techniques are not a substitute for large, clinically curated databases, they provide a complementary route to explore controlled variations in impairment and to probe the robustness of automatic voice pathology detection systems.

Model adaptation is another trend. It uses transfer learning to adapt pretrained models to small clinical corpora [92]. Some studies also use domain adaptation to reduce mismatch across corpora and recording conditions [11]. A further option is to harvest large speech collections from online videos (for example YouTube) for pretraining or augmentation, but these data are not clinically validated, and they show strong channel and noise variability [93].

### 2.5 Introduction to the HUPA corpus

Within this landscape, characterised by a scarcity of openly accessible voice disorder databases, the predominance of small or restricted corpora, and substantial heterogeneity in acquisition and labelling practices, the release of a high-quality corpus in Spanish becomes an important step for advancing research in this area. The limited availability of freely downloadable resources forces researchers to rely on heterogeneous, privately held data, which undermines reproducibility and complicates cross-system comparison. In this context, the HUPA corpus fills a critical gap, as it provides a clinically rich and technically homogeneous data set that has already served for years as a *de facto* reference despite not being publicly released.

In recent years, independent groups have requested HUPA and reused it in multiple studies, even under a restricted request-based access model. This sustained demand highlights both its scientific relevance and the broader need for a Castilian Spanish pathological voice corpus with explicit healthy controls, balanced class composition and well-documented clinical metadata. Making HUPA publicly documented and accessible to *bona fide* researchers under a standard DUA therefore directly addresses structural limitations in the field, particularly the absence of a standardised Spanish benchmark with consistent acquisition conditions, curated annotations and transparent documentation. Its public release transforms an informal reference into a formal and reproducible benchmark, improving methodological consistency and enabling fair and independent external validation, especially in cross-corpora scenarios.

The corpus is intentionally focused: all recordings consist of sustained /a/ vowels captured through a fixed protocol in a single acoustic environment. This design does not aim to compete with large multi-centre registries; instead, it converts a mature single-centre clinical resource into a dataset optimised for sustained vowel analysis, with the corresponding advantages and limitations. Within this scope, HUPA provides homogeneous recording conditions, detailed clinical labelling, explicit healthy controls, balance across clinically relevant categories and sexes, and high-quality perceptual and acoustic metadata. These characteristics help address widespread issues in existing closed corpora, such as limited language coverage, imbalances between classes, and heterogeneous documentation.

In view of the aforementioned, and similarly to the practices followed in other areas of speech technology, corpora accessible to the broader scientific community, as well as standardised metrics to report results, are still required.

Over the past two decades, HUPA has supported a wide range of research efforts, including –with no aim to be exhaustive– methodological analyses of automatic pathology detection [15], studies on compression and transmission effects [16,94], comparisons between acoustic analysis systems [4], feature-space transformations for improved classification [6], multimodal spectral approaches [7], evaluations of feature robustness [5], data-augmentation strategies [8] and systematic reviews for automatic voice-disorder screening systems [17]. Collectively, these studies confirm that HUPA has long operated as a practical reference point for algorithmic evaluation and clinical interpretation, reinforcing the rationale for its open release.

Historically, the corpus has been shared on request with *bona fide* researchers. Despite this restricted-access model, it has been consistently reused by independent groups, which indicates a clear community demand for a well-documented Spanish pathological voice dataset with balanced classes, explicit healthy controls and detailed clinical annotation. Public release therefore represents a major step forward, since it enhances transparency, facilitates direct comparison between methods and enables independent external validation. By making the recordings and metadata openly available with standardised documentation, this work aims to support reproducibility and fair benchmarking in AVCA research.

The present paper has two main goals: **(i)** to provide a concise overview of the dataset and its provenance (participants, recording protocol, equipment and basic quality validation) ensuring its correct reuse; and, **(ii)** to describe how the dataset is made publicly available according to the FAIR principles, its accompanying metadata, and to present some baseline experiments –with no intention to outperform the state of the art–, so HUPA can serve as a benchmark corpus for automatic voice pathology assessment in Castilian Spanish.

The HUPA corpus is publicly documented and distributed through a Zenodo® repository [95] under a standard DUA for non-commercial research use. In summary, HUPA provides a clinically documented and balanced corpus of sustained /a/ vowels recorded using a fixed protocol and professional equipment, ensuring the technical homogeneity necessary to minimize channel variability; detailed clinical labelling and expert perceptual ratings; and curated acoustic parameters and metadata accessible through an open-access repository. As summarised in Table 1, this combination of controlled recording conditions, explicit healthy controls, rich annotation and open availability directly addresses key limitations of existing resources and positions HUPA as a reference benchmark for reproducible research in automatic voice pathology assessment in Castilian Spanish.

**Table 1:** Comparative overview of the most relevant voice disorder databases released following FAIR principles. The imbalance ratio (IR) is defined as the ratio between the number of samples in the majority and minority classes (healthy control vs.\ pathological speakers) [96,97]; values close to 1 indicate a nearly balanced dataset. Participants (ctrl/path)' indicates the number of healthy controls and pathological patients (or recordings). `V': sustained vowel tasks; `S': words or sentences; `CS': connected or continuous speech. `Open (Restricted)' indicates availability via a public repository requiring a DUA. 'On request' indicates access granted via direct contact with authors.

Database	Lang	Speakers (ctrl/path)	IR (maj/min)	Tasks	Access
MEEI	EN	53 / 584	11.0	*V* + *S*	Commercial access
SVD	DE	687 / 1356	2.0	*V* + *S*	Open download
VOICED	IT	58 / 150	2.6	V	Registration required
PVQD	EN	89 / 207	2.3	*V* + *S*	Registration required
UncommonVoice	EN	9 / 48	5.3	*V* + *S*+CS	Controlled access
AVFAD	PT	363 / 346	1.0	*V* + *S*+CS	Controlled access
FEMH (subset)	ZH	50 / 150	3.0	*V* + *S*	Controlled access
MVPD	MS	252 / 130	1.9	V	Controlled access
AVPD	AR	187 / 179	1.0	*V* + *S*+CS	Controlled access
Bridge2AI-Voice (v3.0)	EN	NR / NR	NR	*V* + *S*	Derived data only
Reinke’s edema corpus	ES	30 / 30	1.0	V	Derived data only
HUPA (this work)	ES	239 / 201	1.2	V	Controlled access

## 3 Data Description

**Fig. 1:**
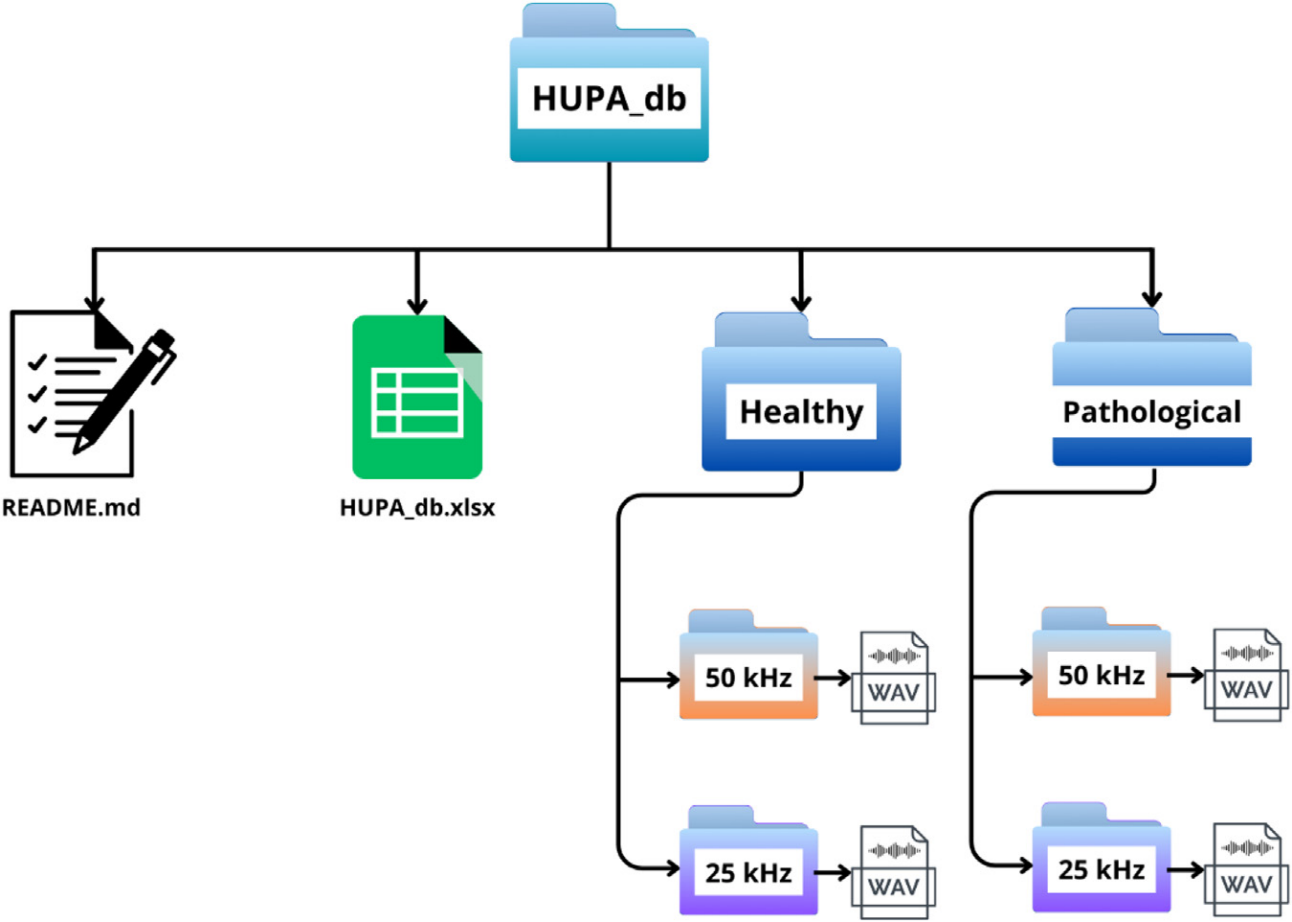
Directory structure of the “HUPA_db” repository, including the metadata files and the organisation of healthy and pathological recordings into “50 kHz” and “25 kHz” subfolders.

Data access is facilitated in a specific Zenodo repository [95]. The HUPA database is distributed in a root folder “HUPA_db” that contains three main elements: **(i)** a human-readable description file (*README.md*); **(ii)** the metadata spreadsheet (*HUPA_db.xlsx*); and **(iii)** two subfolders that store the audio recordings, namely “Healthy” and “Pathological”. The “Healthy” folder contains recordings from healthy control participants, whereas “Pathological” contains recordings from patients with voice disorders. The directory structure of the repository is reported in Fig. 1.

For the sake of compatibility, recordings were converted from the original Normalized Speech File (NSF) format to mono WAV files using the WPCVox package [4]. For each recording, two parallel versions are provided: one sampled at 50 kHz and one at 25 kHz. Both versions represent the same utterance, and no additional denoising, equalisation or dynamic-range processing is applied beyond the acquisition settings described in the Methods section. Within each of the two top-level audio folders (“Healthy” and “Pathological”), the recordings are further organised by sampling frequency into two subfolders: “50kHz” (mono WAV files sampled at 50 kHz, following the standardised naming convention detailed below) and “25kHz” (mono WAV files resampled to 25 kHz, retaining the original filenames used in earlier internal distributions of the corpus). Recordings were trimmed to the steady-state portion of the sustained vowel. We removed the onset and offset so that each released WAV contains only the stable voiced segment. Recordings were trimmed to the steady-state portion of the sustained vowel. We removed the onset and offset so that each released WAV contains only the stable voiced segment.

### 3.1 Naming convention

File names for the 50 kHz recordings follow a structured convention: *patientID_pathologyCode_sex_age_condition*.wav. *patientID* is a numeric identifier; *pathologyCode* identifies the pathology category as listed in Table 2; *sex* is encoded as M (male) or F (female); *age* is given in years; and *condition* takes the value healthy or pathological.

**Table 2:** Pathologies present in the pathological cohort of the HUPA corpus (*n* = 201). For each diagnosis code, the pathology label, participant count, and percentage within the pathological cohort are reported. The column Diagnosis group follows the clinical taxonomy provided in the “Pathology classification” worksheet (Organic pathologies, Associated minimal lesions, or Functional). The column Category provides a simplified clinical label derived from the taxonomy (Structural, Neurogenic, or Functional) to support non clinical users. When a general code is used and more detailed codes also appear in the same table, these related codes are listed under More detailed codes in this table. Counts reflect the cohort composition during the recruitment period and should not be interpreted as population prevalence.

Code	Pathology	Count	%	Diagnosis group	Category	Additional specific codes in this table	Notes
1.1.3	Sulcus	2	1.0	Organic pathologies	Structural	1.1.3.2	Few cases in this release. General code; more specific codes also appear in this table.
1.1.3.2	Sulcus (stria type)	22	10.9	Organic pathologies	Structural		
1.1.4	Epidermoid cyst	20	10.0	Organic pathologies	Structural		
1.2.1.2.1	Iatrogenic lesions (vocal folds)	2	1.0	Organic pathologies	Structural		Few cases in this release.
1.2.2.4	Chronic hyperplastic laryngitis	19	9.5	Organic pathologies	Structural		
1.2.2.5	Chronic hyperplastic laryngitis with leucoplakia	11	5.5	Organic pathologies	Structural		
1.2.5.1	Peripheral paralysis (unspecified side)	1	0.5	Organic pathologies	Neurogenic	1.2.5.1.3, 1.2.5.1.4	Few cases in this release. General code; more specific codes also appear in this table.
1.2.5.1.3	Right recurrent laryngeal nerve paralysis	9	4.5	Organic pathologies	Neurogenic		
1.2.5.1.4	Left recurrent laryngeal nerve paralysis	8	4.0	Organic pathologies	Neurogenic		
1.2.5.2	Upper motor neurone lesion	14	7.0	Organic pathologies	Neurogenic		
1.2.5.3	Extrapyramidal disorders	1	0.5	Organic pathologies	Neurogenic		Few cases in this release.
2.1.2	Bilateral nodule	29	14.4	Associated minimal lesions	Structural		
2.2.1	Pedunculated polyp	28	13.9	Associated minimal lesions	Structural		
2.3.2	Bilateral Reinke’s oedema	29	14.4	Associated minimal lesions	Structural		
3.1.2.1	Incomplete glottal closure	6	3.0	Functional	Functional		
	Total	201	100.0				

### 3.2 Metadata associated with the audio recordings

The metadata for the corpus is consolidated within the *HUPA_db.xlsx* spreadsheet. The file is structured into multiple worksheets to organise the information levels: **(i)** an “Intro” sheet provides a general overview of the dataset, including summary statistics on participant demographics and the distribution of pathologies; **(ii)** the “Pathology classification” sheet serves as a lookup table mapping the numeric Pathology code to specific clinical diagnoses (e.g., mapping code 2.1.2 to “Bilateral nodule”); and **(iii)** the “Healthy” and “Pathological” worksheets contain the core row-level metadata, where each entry lists file identifiers (Patient ID, File name, Original file name), technical specifications (Sampling frequency), demographic variables (Age, Sex), clinical descriptors (Pathology, Pathology code), and consensus perceptual ratings (G, R, B, A, S, Total, as detailed in the 'Perceptual evaluations' section within Experimental Design, Materials and Methods). These worksheets also include basic acoustic estimates.

Formant trajectories (F1–F3) were estimated using the KARMA algorithm [98] The analysis used 20 ms windows and 50 % overlap. An energy based Voice Activity Detection (VAD) was applied with a power quantile threshold of 0.15 to handle non speech frames. VAD is a rule based step that labels each frame as voiced or non voiced, so that silence and unvoiced segments are not used for the estimates. For each sustained vowel, one representative value per formant is reported as the median of the estimated trajectory over the stable portion of the signal.

Fundamental frequency (F0) was estimated with the default AVCA ByO pitch routine [99]. The routine used an autocorrelation based approach. It analysed 40 ms frames with a 20 ms hop, so it used 50 % overlap. It searched pitch periods from 2 ms to 15 ms, which corresponds to about 67 Hz to 500 Hz. It excluded silent frames using the toolbox silence detector, and it kept voiced frames when the normalized autocorrelation peak was above 0.3. One F0 value per recording is reported as the median across voiced frames in the stable portion of the signal.

**Fig. 2:**
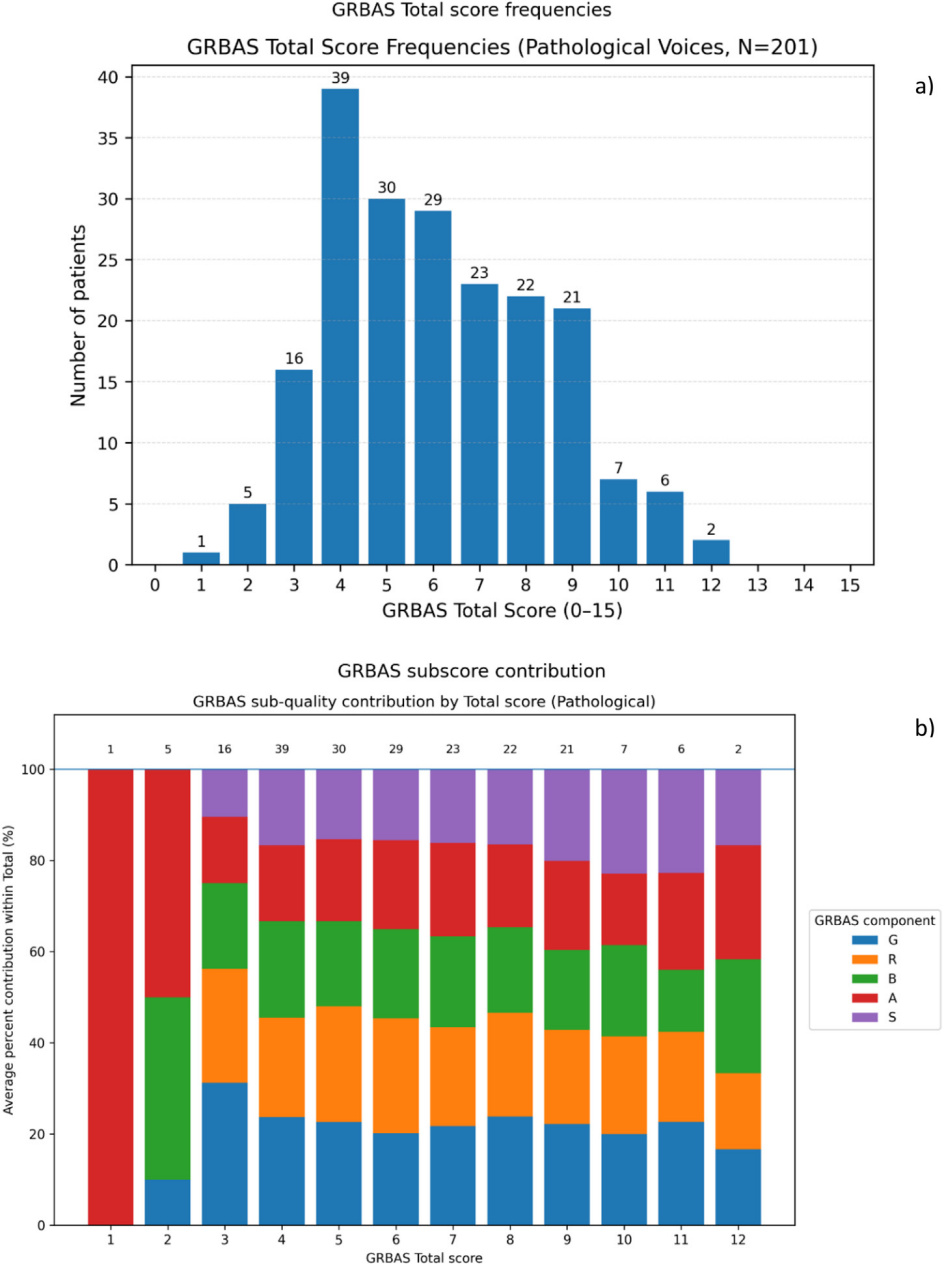
GRBAS severity distribution and sub-score composition in the Pathological group. (a) Frequency of GRBAS Total scores (0–15). (b) Bars stacked to 100 % showing the average percent contribution of the GRBAS sub-scores (G, R, B, A, S) within each Total score. Numbers above bars indicate the number of participants who have that GRBAS score.

The “Formants”, “Peaks”, “Jitter”, and “Comments” columns provide technical notes about signal quality. Finally, the spreadsheet includes two feature worksheets, “Acoustic Features 25 kHz” and “Acoustic Features 50 kHz”. These worksheets store the full set of objective parameters extracted per recording, as described in the “Acoustic Features” section within Experimental Design, Materials and Methods, and they match exactly the list in Table 4. The GRBAS distributions in these worksheets confirm that the corpus covers a clinically relevant range, as shown in Fig. 2.

## 4 Experimental Design, Materials and Methods

### 4.1 Participants

Inclusion criteria for healthy voices were no self-perceived laryngeal or voice problems; voice appropriate for age/sex; non-smoker; no prior surgical intervention; no endotracheal intubation within the previous year; a total GRBAS score strictly below 3; and laryngostroboscopic examination confirming the absence of laryngeal abnormalities [19]. Pathological voices were included when an organic laryngeal lesion was documented in the clinical record. For further details on how GRBAS ratings were obtained, see the **Perceptual evaluations subsection**.

The corpus comprises 440 native Castilian Spanish participants, divided into healthy controls and pathological cohorts. Each recording corresponds to a unique patient. The detailed demographic breakdown by sex and group is provided in Table 3.

**Table 3:** Participants demographics by group and sex.

Group	Male	Female	Total
Pathological	74	127	201
Healthy Control	101	138	239
Overall	175	265	440

The dataset includes a balanced cohort which was developed for screening purposes (239 healthy controls and 201 pathological patients), with a sex distribution reflecting the epidemiological balance (41,8 % men and 58,2 % women) [100].

### 4.2 Speech task and protocol

Each participant produced a sustained Castilian Spanish vowel /a/ with a comfortable pitch and loudness. Participants were seated in a quiet clinical room; a speech-language pathologist monitored the task and selected the most stable utterance for inclusion.

### 4.3 Recording setup

Recordings were carried out at HUPA (Alcalá de Henares, Madrid) in collaboration with Universidad Politécnica de Madrid (UPM), following identical conditions for all participants. Signals were acquired with a Kay Elemetrics CSL® 4300B system at a sampling rate of 50 kHz, with 16-bit quantisation, using Pulse Code Modulation (PCM) coding, and originally stored in the proprietary NSF file format. A cardioid dynamic Shure® SM48 vocal microphone was placed at a fixed distance of approximately 15 cm from the mouth and at an angle of approximately 45 degrees. The effective bandwidth of the recordings is limited to about 14 kHz, in accordance with the nominal frequency response of the microphone. Input gain was fixed, and no automatic processing such as Automatic Gain Control (AGC) or noise reduction was applied. All recordings are mono and have a duration of approximately 2 s. Sessions were conducted in an acoustically treated–but not fully isolated–room, with reverberation time measured below 0.25 s and background noise below 40 dB SPL.

This recording protocol follows common practice in clinical voice research. Standardised recording conditions help comparability across studies because acoustic measures can change with the microphone, the recording interface, and the environment [101,102]. Similar controlled recording protocols have been recommended for instrumental voice assessment, and similar microphone placement has been reported in prior acoustic studies [103,104].

### 4.4 Perceptual evaluations

Perceptual severity was evaluated using the GRBAS scale on a four-point ordinal scale from 0 (normal) to 3 (severe), yielding subscores G, R, B, A, and S, and a Total score from 0 to 15 [19]. All participants were rated in situ by a single otolaryngologist (ENT) who is currently affiliated with Hospital Universitario de Fuenlabrada (Madrid, Spain). One final ordinal score per GRBAS subscore was assigned for each participant. The GRBAS scale has been reported to show acceptable reliability [105] and correlations with acoustic markers [55].

### 4.5 Acoustic features

For each recording, a set of acoustic features was pre-computed using the AVCA-ByO toolbox [99]. The feature set covers four main domains: noise measures, perturbation measures, tremor characteristics, and complexity features relevant for voice-quality assessment [106]. The default toolbox settings were used. The toolbox applied peak normalization. For the noise, perturbation, and tremor characteristics, it resamples the signal to 25 kHz if needed. It used 40 ms frames and 50 percent overlap.

For **perturbation measures and tremor characteristics**, the toolbox computed an internal F0 estimate with the Kasuya Feijoo method. This F0 estimate was used only as an intermediate step to derive the final perturbation measures and tremor characteristics. For **tremor characteristics**, it low pass filtered the signal at 30 Hz, it downsampled the signal to about 400 Hz, and it searched tremor frequencies from 2 Hz to 10 Hz.

For **noise measures**, the toolbox used the same frame length and overlap. For GNE, it used a 40 ms window, a bandwidth of 500 Hz, and a band shift of 100 Hz. For **complexity features**, the toolbox used 40 ms frames with a Hamming window and 50 percent overlap. It used an embedding dimension of 8 and a delay of 20 samples. This delay depends on the sampling rate. The toolbox did not apply silence detection in this block by default. The complete list of extracted parameters is provided in Table 4.

**Table 4:** Acoustic parameters extracted from the recordings using the AVCA-ByO toolbox [99].

Abbreviation	Parameter	Unit
Noise Parameters		
HNR	Harmonics-to-Noise Ratio	dB
CHNR	CepstrumHarmonics-to-Noise Ratio	dB
GNE	Glottal-to-Noise Excitation Ratio	Ratio
NNE	Normalised Noise Energy	dB
Perturbation Measures		
CPP	Cepstral Peak Prominence	dB
ShimmerDb	Absolute Shimmer	dB
rShimmer	Relative Shimmer	%
APQ	Amplitude Perturbation Quotient	%
sAPQ	Smoothed Amplitude Perturbation Quotient	%
Jitter	Absolute Jitter	μs
rJitter	Relative Jitter	%
RAP	Relative Average Perturbation	%
rPPQ	Pitch Period Perturbation Quotient	%
rSPPQ	Smoothed Pitch Period Perturbation Quotient	%
Tremor Parameters		
FTRI	Frequency Tremor Intensity Index	Arbitrary Units
ATRI	Amplitude Tremor Intensity Index	Arbitrary Units
FFTR	Fundamental Frequency Tremor Frequency	Hz
ATRF	Amplitude Tremor Frequency	Hz
Complexity Measures		
ApEn	Approximate Entropy	Arbitrary Units
SampEn	Sample Entropy	Arbitrary Units
FuzzyEn	Fuzzy Entropy	Arbitrary Units
GSampEn	Generalised Sample Entropy	Arbitrary Units
mSampEn	Multiscale Sample Entropy	Arbitrary Units
CorrDim	Correlation Dimension	Dimensionless
LLE	Largest Lyapunov Exponent	Dimensionless
Hurst	Hurst Exponent	Dimensionless
mDFA	Multifractal Detrended Fluctuation Measure	Arbitrary Units
RPDE	Recurrence Period Density Entropy	Arbitrary Units
PE	Permutation Entropy	Arbitrary Units
MarkEnt	Markov Entropy	Arbitrary Units

A short note on comparability is relevant because these feature domains are widely used in automatic voice condition analysis and have been explored across many pathological voice studies and datasets [99].

### 4.6 Technical validation

**Fig. 3:**
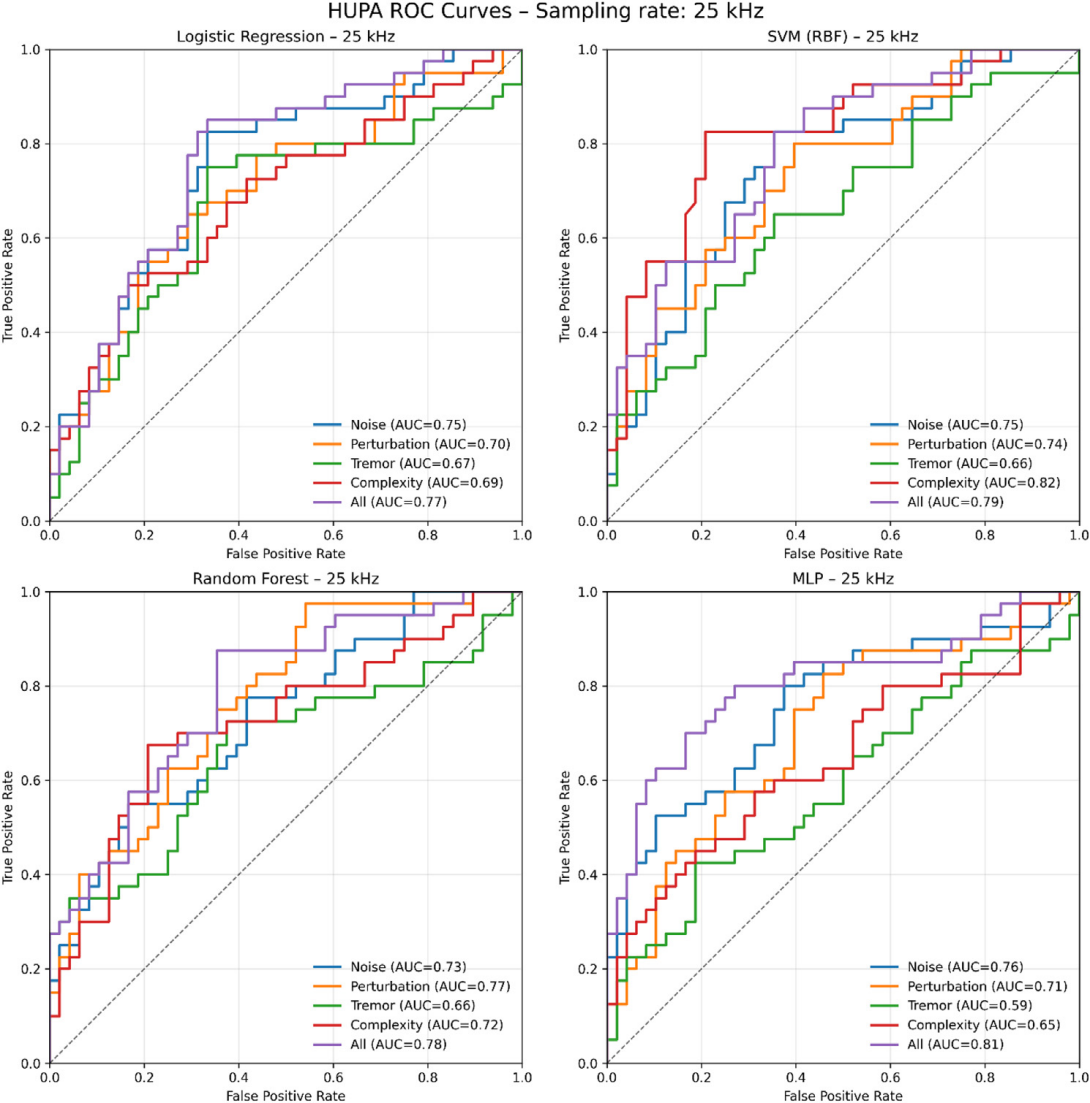
ROC curves on the held-out test set (25 kHz). Each subplot corresponds to one classifier (Logistic Regression, SVM-RBF, Random Forest, and MLP). Within each subplot, ROC curves are shown for each feature set (noise, perturbation, tremor, complexity, and all features).

The dataset includes GRBAS subscores (G, R, B, A, S) and the corresponding Total score. Internal consistency was checked, and the Total score equals the sum of the five subscores for all entries. In the Pathological group (*N* = 201), Total scores range from 1 to 12, with a median of 6 (IQR 4 to 8) (Fig. 2a). This shows that the dataset includes many voices with clear deviation, but it is not limited to only very severe cases. In the Healthy group (*N* = 239), Total scores range from 0 to 4, with a median of 0 (IQR 0 to 1), so most controls were rated as normal or very close to normal. Small values above zero in Healthy are expected in real data. Perceptual ratings have normal variability, and some speakers can show slight deviations even without a diagnosis. Fig. 3.

In the Pathological group, the mean ± SD per subscore was G 1.36±0.60, R 1.36±0.72, B 1.17±0.67, A 1.16±0.76, and S 1.04±0.81. Fig. 2b adds detail by showing how the Total score is formed. For the same Total score, the value can come mainly from one high subscore, or from several medium subscores. In HUPA, G and R contribute more on average than the other subscores, while S contributes the least. This suggests that roughness and overall grade are more common than strain in this cohort. Sex specific descriptive statistics (mean ± SD) are reported in Table 5.

**Table 5:** Sex-specific GRBAS descriptive statistics (mean ± SD). (Healthy: Female *n* = 138, Male *n* = 101; Pathological: Female *n* = 127, Male *n* = 74).

Group	Sex	No. participants	G	R	B	A	S	Total
Healthy	Female	138	0.04 ± 0.19	0.08 ± 0.27	0.04 ± 0.20	0.34 ± 0.51	0.08 ± 0.27	0.58 ± 0.82
Healthy	Male	101	0.05 ± 0.22	0.19 ± 0.44	0.02 ± 0.14	0.20 ± 0.42	0.03 ± 0.17	0.49 ± 0.77
Pathological	Female	127	1.40 ± 0.61	1.35 ± 0.73	1.23 ± 0.66	1.22 ± 0.80	1.12 ± 0.79	6.31 ± 2.35
Pathological	Male	74	1.30 ± 0.59	1.39 ± 0.70	1.07 ± 0.69	1.05 ± 0.68	0.91 ± 0.83	5.72 ± 2.21

To assess the internal consistency and discriminative power of the features, a supervised classification experiment (healthy vs. pathological) was conducted. The descriptors were grouped into four blocks (noise, perturbation, tremor, complexity), and an additional combined set including all features was also evaluated. An 80/20 stratified split was used. Four standard classifiers were evaluated: Logistic Regression, Support Vector Machine (SVM) with a Radial Basis Function (RBF) kernel, Random Forest, and Multilayer Perceptron (MLP). On the 25 kHz data, the best-performing classifiers achieved test-set Area Under the Curve (AUC) values of 0.76 for noise features, 0.77 for perturbation measures, 0.67 for tremor parameters, and 0.82 for complexity measures. When all feature blocks were combined into a single set, the MLP achieved the best test performance, with a test AUC of 0.81.

For the complexity block, the best model was SVM-RBF (test AUC = 0.82; CV AUC = 0.78). For the combined all-features set, the best model was MLP (test AUC = 0.81). Sex-stratified AUC values were computed both on the held-out test set and using out-of-fold (OOF) predictions across the full dataset. Decision thresholds were selected from OOF predictions using Youden’s J, and the corresponding test confusion-matrix metrics are reported. Table 6 summarises the sex-stratified AUC results (test and OOF), the OOF thresholds, and the test confusion-matrix metrics for both models**.** A subtype-level error audit was computed using OOF predictions across the full 25 kHz dataset. Table 7 reports only the top five pathology subtypes with the highest OOF false-negative counts.

**Table 6:** Performance summary for the best 25 kHz models using the Complexity feature block (SVM-RBF) and the combined All-features set (MLP). The table reports test-set AUC and cross-validation (CV) AUC. Sex-stratified AUC values are given both for the held-out test set and for OOF predictions computed across the full dataset (each sample is evaluated in a fold where it was not used for training). The decision threshold was selected from the OOF predictions using Youden’s J statistic (maximising sensitivity + specificity − 1). Confusion-matrix metrics are computed on the held-out test set using that threshold, including sensitivity (true-positive rate), specificity (true-negative rate), and balanced accuracy (mean of sensitivity and specificity). TP, FN, TN, and FP denote the numbers of true positives, false negatives, true negatives, and false positives in the test split, where the positive class corresponds to pathological recordings and the negative class to healthy recordings.

Feature set	Model	Test AUC	CV AUC	Youden (OOF)	Sens	Spec	BalAcc	Test AUC (F)	Test AUC (M)	OOF AUC (F)	OOF AUC (M)
CPLX	SVM-RBF	0.82	0.78	0.51	0.65	0.83	0.74	0.81	0.81	0.78	0.79
ALL	MLP	0.81	0.81	0.38	0.75	0.75	0.75	0.79	0.83	0.80	0.84

**Table 7:** Top five pathology subtypes with the highest number of OOF false negatives in the 25 kHz dataset. Results are reported for the best-performing model trained on the Complexity feature block (SVM with RBF kernel) and for the best-performing model trained on the combined All-features set (MLP). N pathological (OOF total) is the number of pathological recordings available for each subtype in the full dataset. FN (Complexity SVM-RBF) and FN (All-features MLP) are the numbers of pathological recordings from that subtype that were predicted as healthy (false negatives) using OOF predictions. FN rate is the subtype-specific false-negative rate, computed as FN divided by N pathological (OOF total). FN (Total) is the sum of false negatives across the two models and is used to rank the subtypes in this table. Pathology subtypes are identified by their numeric Pathology code and the corresponding hierarchical label (Full pathology name).

Pathology code	Full pathology name	No. pathological (OOF total)	FN (Complexity SVM-RBF)	FN rate (Complexity)	FN (All-features MLP)	FN rate (All-features)	FN (Total)
221	Associated minimal lesions > Polyp > Pedunculated	28	11	0.393	6	0.214	17
212	Associated minimal lesions > Nodule > Bilateral	29	6	0.207	6	0.207	12
1132	Organic pathologies > Congenital > Sulcus > Stria sulcus	22	7	0.318	5	0.227	12
114	Organic pathologies > Congenital > Epidermoid cyst	20	7	0.350	4	0.200	11
232	Associated minimal lesions > Reinke's oedema > Bilateral	29	5	0.172	5	0.172	10

### 4.7 External validation

HUPA has been used in many peer reviewed studies on feature design, deep representations, and cross corpus transfer. This is expected because HUPA shares key elements with several other voice disorder corpora reported in the Background section. Many datasets include adult speakers and sustained vowel tasks, and they support a healthy control versus pathological setting. These common constraints make cross corpus experiments more meaningful.

Early work on acoustic descriptors already treated HUPA as a challenging benchmark. In particular, [7,12,18] exploited modulation spectrum (MS) features, either alone or combined with Mel-Frequency Cepstral Coefficients (MFCC), and reported strong in-database performance on HUPA as well as meaningful cross-database generalisation between MEEI and HUPA when using appropriate MS normalisation and feature fusion.

Building on this line, [13] systematically tuned a family of MS-derived metrics on both MEEI and HUPA, showing that optimised MS parameters can reach competitive detection efficiencies on HUPA and, importantly, that the optimal parameter ranges are largely consistent across corpora. Complementary to these studies focused on specific descriptors, [5] proposed a complex parameterisation that aggregates more than 90 conventional, non-linear and modulation-based features and evaluated it on three databases (MEEI, HUPA and Czech Parkinsonian Speech Database (PARCZ)), highlighting HUPA as a demanding but informative testbed for large feature sets.

On the modelling side, HUPA has been repeatedly used in multi-database experimental setups. [107] investigated supervised deep learning (DenseNet on raw waveforms and spectrograms), gradient boosting (XGBoost on dysphonic features) and anomaly detection (Isolation Forest) across AVPD, MEEI, HUPA and SVD, using the sustained /a/ and careful chunking and re-weighting to address class imbalance. Their results confirmed that HUPA can be integrated into large heterogeneous benchmarks without collapsing performance.

A complementary body of work focusses on glottal and source-related information. [3] estimated the glottal volume velocity waveform via quasi-closed-phase inverse filtering and parametrised it with temporal, spectral and LF-model descriptors, performing extensive intra- and cross-database experiments on AVPD, MEEI, SVD and HUPA. They reported that glottal-flow features provide effective voice-disorder detection and severity assessment on HUPA and remain robust under cross-corpus training. More recently, [2] evaluated glottal-source features on HUPA and SVD, finding performance comparable to or better than MFCC and Perceptual Linear Prediction (PLP) alone and best when source and tract cues are combined, further supporting the use of HUPA to study source–tract complementarity. From a different angle, [1] proposed low-dimensional energy and statistical features and tested them with SVM and Stochastic Gradient Descent (SGD) on SVD, HUPA, AVPD and MEEI, showing that relatively simple descriptors can still achieve high accuracies on HUPA in both binary and multiclass settings.

Representation choice and deep feature learning have also been explicitly addressed on HUPA. On the input side, [10] compared 1-D spectral features (e.g., MFCC, RelAtive SpecTrAl PLP - RASTA-PLP)) with 2-D time–frequency inputs on HUPA and reported that a 2-D CNN trained on dynamic MFCC spectrograms achieved 81 % accuracy, outperforming the strongest 1-D baselines. From a cross-corpus perspective, [11] introduced Joint Subspace Transfer Learning (JSTL) and reported strong cross-database accuracies across MEEI, SVD and HUPA with gains up to ∼15 percentage points over prior transfer methods. Earlier cross-database evidence was already provided in [1,3,12,13,107], which consistently shows that systems tuned on MEEI or other corpora can retain useful discrimination when evaluated on HUPA and vice versa.

Finally, robustness to realistic clinical noise has been evaluated in [14], where the authors assessed asymmetric multi-condition training (MCT) using four databases, including HUPA. On the HUPA (polyps) subset, their random-forest baseline moved from strong clean performance to robust results under asymmetric training, supporting MCT as a conservative strategy that preserves performance without leakage.

### 4.8 Code availability and usage notes

To facilitate the reproducibility of the results and the usage of the HUPA database, the complete analysis pipeline presented in the previous section has also been made publicly available in a GitHub® repository (https://github.com/BYO-UPM/HUPA_Database). This repository contains the source code required to load the feature data, perform data preprocessing, and execute the binary classification task (Pathological vs. Healthy Control).

### 4.9 The workflow is divided into three specific scripts

• **HUPA_Features_Extraction.m (MATLAB):** This script performs acoustic feature extraction. It relies on the AVCA-ByO toolbox [99] to calculate the features described in the Acoustic Features section.
• **HUPA_PRN_GridSearch_ROC.m (MATLAB):** This script executes classification analysis within the MATLAB environment. It implements a Grid Search with Cross-Validation (CV) to optimise the hyperparameters of distinct models (Logistic Regression, SVM, Random Forest, and MLP) and evaluates the best models using ROC analysis on a hold-out test set.
• **HUPA_Python_GridSearch.py (Python):** This script offers a parallel implementation of the machine learning pipeline using the scikit-learn [108] library. It mirrors the functionality of the MATLAB classification script–including data splitting, feature grouping, grid search optimisation, and performance evaluation–to ensure accessibility for Python users.

## Limitations

The HUPA corpus is restricted to sustained /a/ vowels and does not include continuous speech tasks (such as reading passages or spontaneous speech), which limits the analysis of prosodic and articulatory features. Additionally, the data was collected at a single centre (HUPA), which ensures high technical homogeneity but may limit the diversity of background acoustic conditions compared to multi-centre studies. The effective bandwidth of the recordings is limited to approximately 14 kHz due to the microphone characteristics. Finally, although the dataset presents a balanced distribution between control and pathological groups, the prevalence of specific disorders within the pathological cohort is uneven (intra-class imbalance), resulting in some categories having a limited sample size.

## Ethics Statement

The open publication of the HUPA corpus was reviewed by the Ethics Committee of UPM (Ref. CE251128). The dataset contains fully anonymised voice recordings collected with informed consent, and no personal identifiers are included in the public release. All procedures comply with the principles of the Declaration of Helsinki and the EU General Data Protection Regulation (GDPR 2016/679).

## CRediT Author Statement

**Juan C. Puerta-Acevedo:** Validation, Writing - Original Draft, Software. **Maria F. Alcala-Durand:** Validation, Review & Editing. **Janaína Mendes-Laureano:** Data curation, Investigation (labelling data). **Julián D. Arias-Londoño:** Methodology, Review & Editing, Supervision. **Juan I. Godino-Llorente:** Conceptualisation, Data Curation, Methodology, Resources, Writing - Original Draft, Supervision, Project administration, Funding acquisition.

## Appendix Supplementary materials

Image, application 1

Image, application 2

## References

[1] Shrivas A., Deshpande S., Gidaye G., Nirmal J., Ezzine K., Frikha M., Desai K., Shinde S., Oza A.D., Burduhos-Nergis D.D., Burduhos-Nergis D.P. (2022). Employing energy and statistical features for automatic diagnosis of voice disorders. Diagnostics.

[2] Kadiri S.R., Alku P. (2020). Analysis and detection of pathological voice using glottal source features. IEEe J. Sel. Top. Signal. Process..

[3] Gidaye G., Nirmal J., Ezzine K., Shrivas A., Frikha M. (2020). Application of glottal flow descriptors for pathological voice diagnosis. Int. J. Speech. Technol..

[4] Godino-Llorente J.I., Osma-Ruiz V., Sáenz-Lechón N., Cobeta-Marco I., González-Herranz R., Ramírez-Calvo C. (2008). Acoustic analysis of voice using WPCVox: a comparative study with Multi Dimensional Voice Program. European Archives of Oto-Rhino-Laryngology.

[5] Mekyska J., Janousova E., Gomez-Vilda P., Smekal Z., Rektorova I., Eliasova I., Kostalova M., Mrackova M., Alonso-Hernandez J.B., Faundez-Zanuy M., López-de-Ipiña K. (2015). Robust and complex approach of pathological speech signal analysis. Neurocomputing..

[6] Arias-Londoño J.D., Godino-Llorente J.I., Sáenz-Lechón N., Osma-Ruiz V., Castellanos-Domínguez G. (2010). An improved method for voice pathology detection by means of a HMM-based feature space transformation. Pattern. Recognit..

[7] Arias-Londoño J.D., Godino-Llorente J.I., Markaki M., Stylianou Y. (2011). On combining information from modulation spectra and mel-frequency cepstral coefficients for automatic detection of pathological voices. Logopedics Phoniatrics Vocology.

[8] Javanmardi F., Kadiri S.R., Alku P. (2024). A comparison of data augmentation methods in voice pathology detection. Comput. Speech Language.

[9] Harar P., Alonso-Hernandez J.B., Mekyska J., Galaz Z., Burget R., Smekal Z. (2017). Proceedings of the International Work-Conference on Bio-Inspired Intelligence.

[10] Javanmardi F., Kadiri S.R., Kodali M., Alku P. (2022). Proceedings of the Interspeech Conference.

[11] Zhang Y., Qian J., Zhang X., Xu Y., Tao Z. (2022). Pathological voice detection using Joint Subspace Transfer learning. Appl. Sci. (Switzerland).

[12] Markaki M., Stylianou Y. (2009). Proceedings of the Interspeech Conference.

[13] Moro-Velázquez L., Gómez-García J.A., Godino-Llorente J.I. (2016). Voice pathology detection using modulation spectrum-optimized metrics. Front. Bioeng. Biotechnol..

[14] Madruga M., Campos-Roca Y., Perez C.J. (2021). Multicondition training for noise-robust detection of benign vocal fold lesions from recorded speech. IEEe Access..

[15] Sáenz-Lechón N., Godino-Llorente J.I., Osma-Ruiz V., Gómez-Vilda P. (2006). Methodological issues in the development of automatic systems for voice pathology detection. Biomed. Signal. Process. Control.

[16] Sáenz-Lechón N., Osma-Ruiz V., Godino-Llorente J.I., Blanco-Velasco M., Cruz-Roldán F., Arias-Londoño J.D. (2008). Effects of audio compression in automatic detection of voice pathologies. IEEE Trans. Biomed. Eng..

[17] Nudelman C.J., Tardini V., Bottalico P. (2025). Artificial intelligence to detect voice disorders: an AI-supported systematic review of accuracy outcomes. J. Voice.

[18] Markaki M., Stylianou Y., Arias-Londoño J.D., Godino-Llorente J.I. (2010). Proceedings of the IEEE International Conference on Acoustics, Speech, and Signal Processing.

[19] Hirano M., McCormick K.R. (1981).

[20] Gómez-García J.A., Moro-Velázquez L., Godino-Llorente J.I. (2019). On the design of automatic voice condition analysis systems. Part I: review of concepts and an insight to the state of the art. Biomed. Signal. Process. Control.

[21] Ezzahori H., Hammimou A., Boudaoud A., Aqil M. (2025). Machine Learning Approaches to laryngeal pathologies detection and classification: a comprehensive literature review. Int. J. Bioautom..

[22] Fourcin A., Abberton E. (1998). Proceedings of the Laryngeal Imaging and Voice Analysis Workshop.

[23] Hirschberg J., Hjalmarsson A., Elhadad N., Neusteun A. (2010). Advances in Speech Recognition.

[24] Stasak B., Epps J., Lawson A. (2018). Proceedings of the 17th Australasian Speech Science and Technology.

[25] Stasak B., Epps J., Larsen M., Christensen H. (2022). Proceedings of the 17th Australasian Conference on Speech Science and Technology.

[26] Ibarra E.J., Arias-Londoño J.D., Zañartu M., Godino-Llorente J.I. (2023). Towards a corpus (and Language)-independent screening of Parkinson’s disease from voice and speech through domain adaptation. Bioengineering.

[27] Lippmann R.P. (1997). Speech recognition by machines and humans. Speech. Commun..

[28] Wilkinson M.D., Dumontier M., Aalbersberg Ij.J., Appleton G., Axton M., Baak A., Blomberg N., Boiten J.W., da Silva Santos L.B., Bourne P.E., Bouwman J., Brookes A.J., Clark T., Crosas M., Dillo I., Dumon O., Edmunds S., Evelo C.T., Finkers R., Gonzalez-Beltran A., Gray A.J.G., Groth P., Goble C., Grethe J.S., Heringa J., t Hoen P.A.C., Hooft R., Kuhn T., Kok R., Kok J., Lusher S.J., Martone M.E., Mons A., Packer A.L., Persson B., Rocca-Serra P., Roos M., van Schaik R., Sansone S.A., Schultes E., Sengstag T., Slater T., Strawn G., Swertz M.A., Thompson M., Van Der Lei J., Van Mulligen E., Velterop J., Waagmeester A., Wittenburg P., Wolstencroft K., Zhao J., Mons B. (2016). The FAIR Guiding Principles for scientific data management and stewardship. Sci. Data.

[29] Evangelista E., Kale R., McCutcheon D., Rameau A., Gelbard A., Powell M., Johns M., Law A., Song P., Naunheim M., Watts S., Bryson P.C., Crowson M.G., Pinto J., Bensoussan Yael E., Olivier E., Anaïs R., Alexandros S., Satrajit G., Powell Maria E., Alistair J., Vardit R., Jean-Christophe B.P., David D., Phillip P., Bensoussan Y. (2024). Current practices in voice data collection and limitations to voice AI research: a national survey. Laryngoscope.

[30] European Parliament, Council of the European Union, Regulation (EU) 2016/679 of the European Parliament and of the Council of 27 April 2016 on the protection of natural persons with regard to the processing of personal data and on the free movement of such data, and repealing Directive 95/46/EC (General Data Protection Regulation), 2016. https://eur-lex.europa.eu/legal-content/EN/TXT/?uri=CELEX:32016R0679.

[31] Van Panhuis W.G., Paul P., Emerson C., Grefenstette J., Wilder R., Herbst A.J., Heymann D., Burke D.S. (2014). A systematic review of barriers to data sharing in public health. BMC. Public Health.

[32] The Office for Civil Rights (OCR), B. Malin, guidance regarding methods for de-identification of protected health information in accordance with the Health Insurance Portability and Accountability Act (HIPAA) Privacy Rule, 2012.

[33] Hartman T., Howell M.D., Dean J., Hoory S., Slyper R., Laish I., Gilon O., Vainstein D., Corrado G., Chou K., Po M.J., Williams J., Ellis S., Bee G., Hassidim A., Amira R., Beryozkin G., Szpektor I., Matias Y. (2020). Customization scenarios for de-identification of clinical notes. BMC. Med. Inform. Decis. Mak..

[34] Tryka K.A., Hao L., Sturcke A., Jin Y., Wang Z.Y., Ziyabari L., Lee M., Popova N., Sharopova N., Kimura M., Feolo M. (2014). NCBI’s database of genotypes and phenotypes: dbGaP. Nucleic Acids Res..

[35] Lappalainen I., Almeida-King J., Kumanduri V., Senf A., Spalding J.D., Ur-Rehman S., Saunders G., Kandasamy J., Caccamo M., Leinonen R., Vaughan B., Laurent T., Rowland F., Marin-Garcia P., Barker J., Jokinen P., Torres A.C., De Argila J.R., Llobet O.M., Medina I., Puy M.S., Alberich M., De La Torre S., Navarro A., Paschall J., Flicek P. (2015). The European Genome-phenome Archive of human data consented for biomedical research. Nat. Genet..

[36] Knoppers B.M. (2014). Framework for responsible sharing of genomic and health-related data. Human Genome Organisation.

[37] Van de Heyning P.H., Remacle M., Van Cauwenberge P., De Bodt M., Wuyts F.L., Moerman M., Van Lierde K., Raes J., Heylen L., Sasserath M., De Hassonville A.H., Clement P.A.R., Qiu J., Molenberghs G., Bruckers L., Mertens F., Pattyn J., Millet B. (1996). Research work of the Belgian Study Group on Voice disorders. Acta Otorhinolaryngol. Belg..

[38] A. Ghio, G. Pouchoulin, F. Viallet, A. Giovanni, V. Woisard, L. Crevier-Buchman, F. Hirsch, C. Fauth, C. Fredouille, Du recueil à l’exploitation des corpus de parole « pathologique » : comment accéder à la variation physiopathologique?, Corpus (2021).

[39] Hariharana M., Polatb K., Sindhuc R., Yaacoba S. (2013). A hybrid expert system approach for telemonitoring of vocal fold pathology. Appl. Soft. Comput..

[40] Ritchings R.T., McGillion M., Moore C.J. (2002). Pathological voice quality assessment using artificial neural networks. Medical Eng. Phys..

[41] Reroń E., Tadeusiewicz R., Modrzejewski M., Wszołek W. (1998). Application of neural networks and pattern recognition methods to the evaluation of speech deformation degree for patients surgically treated for larynx cancer. Neuroendocrinol Lett.

[42] Kuo J., Holmberg E.B., Hillman R.E. (1999). Proceedings of the IEEE International Conference on Acoustics, Speech, and Signal Processing.

[43] Rosa M., Pereira J.C., Grellet M. (2000). Adaptive estimation of residue signal for voice pathology diagnosis. IEEE Trans. Biomed. Eng..

[44] Rosa M., Pereira J.C., Greller M., Carvalho A.C.P.L.F. (1999). Proceedings of the 6th IEEE International Conference on Electronics Circuits Systems.

[45] Alonso J.B., De Leon J., Alonso I., Ferrer M.A. (2001). EURASIP Journal on Applied Signal Processing 2001.

[46] Uloza V., Saferis V., Uloziene I. (2005). Perceptual and acoustic assessment of voice pathology and the efficacy of endolaryngeal phonomicrosurgery. J. Voice.

[47] Ma E.P.M., Yiu E.M.L. (2006). Multiparametric evaluation of dysphonic severity. J. Voice.

[48] Gelzinis A., Verikas A., Bacauskiene M. (2008). Automated speech analysis applied to laryngeal disease categorization. Comput. Methods Programs Biomed..

[49] Vaiciukynas E., Verikas A., Gelzinis A., Bacauskiene M., Uloza V. (2012). Exploring similarity-based classification of larynx disorders from human voice. Speech. Commun..

[50] Wang J., Jo C. (2006). Proceedings of the 11th Australasian International Conference on Speech Science and Technology.

[51] Vasilakis M., Stylianou Y. (2009). Voice pathology detection based on short-term jitter estimations in running speech. Folia Phoniatrica et Logopaedica.

[52] Gómez-Vilda P., Fernández-Baillo R., Rodellar-Biarge V., Lluis V.N., Álvarez-Marquina A., Mazaira-Fernández L.M., Martínez-Olalla R., Godino-Llorente J.I. (2009). Glottal source biometrical signature for voice pathology detection. Speech. Commun..

[53] Péan V., Ouayoun M., Fugain C., Meyer B., Chouard C.H. (2000). A fractal approach to normal and pathological voices. Acta Otolaryngologica.

[54] Paniagua M.S., Pérez C.J., Calle-Alonso F., Salazar C. (2020). An acoustic-signal-based preventive program for university lecturers’ Vocal health. Journal of Voice.

[55] Bhuta T., Patrick L., Garnett J.D. (2004). Perceptual evaluation of voice quality and its correlation with acoustic measurements. Journal of Voice.

[56] Demenko G., Obrebowski A., Pruszewicz A., Wiskirska-Woznica B., Swidzinski P., Wojnowski W. (2004). Proceedings of the Speech Prosody Conference 2004.

[57] Pouchoulin G., Fredouille C., Bonastre J.E., Ghio A., Giovanni A. (2007). Proceedings of the Interspeech Conference.

[58] Alpan A., Maryn Y., Kacha A., Grenez F., Schoentgen J. (2011). Multi-band dysperiodicity analyses of disordered connected speech. Speech. Commun..

[59] Madruga M., Campos-Roca Y., Pérez C.J. (2021). Impact of noise on the performance of automatic systems for vocal fold lesions detection. Biocybernetics and Biomedical Engineering.

[60] Kukharchik P., Martynov D., Kheidorov I., Kotov O. (2007). Proceedings of the 15th European Signal Processing Conference.

[61] Kukharchik P., Kheidorov I., Bovbel E., Ladeev D. (2008). Proceedings of the International Conference on Image and Signal Processing.

[62] Hakkesteegt M.M., Brocaar M.P., Wieringa M.H., Feenstra L. (2008). The relationship between perceptual evaluation and objective multiparametric evaluation of dysphonia severity. J. Voice.

[63] Kim G.H., Lee Y.W., Bae I.H., Park H.J., Wang S.G., Kwon S.B. (2019). Validation of the Acoustic Voice Quality Index in the korean language. Journal of Voice.

[64] Hosokawa K., Barsties B., Iwahashi T., Iwahashi M., Kato C., Iwaki S., Sasai H., Miyauchi A., Matsushiro N., Inohara H., Ogawa M., Maryn Y. (2017). Validation of the Acoustic Voice Quality Index in the japanese language. J. Voice.

[65] M. Pützer, W.J. Barry, Saarbrücken Voice Database, (2008). https://zenodo.org/records/16874898 (accessed January 14, 2026).

[66] Cesari U., De Pietro G., Marciano E., Niri C., Sannino G., Verde L. (2018). Computers and Electrical Engineering.

[67] Walden P.R. (2022). Perceptual voice qualities database (PVQD): database characteristics. J. Voice.

[68] Moore M., Papreja P., Saxon M., Berisha V., Panchanathan S. (2020). Proceedings of the Interspeech Conference.

[69] Jesus L.M.T., Belo I., Machado J., Hall A., Miranda Fernandes F.D. (2017). Advances in Speech-Language Pathology.

[70] Ramalingam A., Kedari S., Vuppalapati C. (2018). Proceedings of the IEEE International Conference on Big Data.

[71] Arias-Londoño J.D., Gómez-García J.A., Moro-Velazquez L., Godino-Llorente J.I. (2018). Proceedings of the IEEE International Conference on Big Data.

[72] Bhat C., Kopparapu S.K. (2018). Proceedings of the IEEE International Conference on Big Data.

[73] Hung C.H., Wang S.S., Te Wang C., Fang S.H. (2022). Using SincNet for learning pathological voice disorders. Sensors.

[74] Fang S.H., Te Wang C., Chen J.Y., Tsao Y., Lin F.C. (2019). Combining acoustic signals and medical records to improve pathological voice classification. APSIPA Trans. Signal Inf. Process..

[75] Wang S.S., Te Wang C., Lai C.C., Tsao Y., Fang S.H. (2022). Continuous speech for improved learning pathological voice disorders. IEEE Open J. Eng. Med. Biol..

[76] Za’im N.A.N., AL-Dhief F.T., Azman M., Alsemawi M.R.M., Abdul Latiff N.M., Baki M.Mat (2023). The accuracy of an Online Sequential Extreme Learning Machine in detecting voice pathology using the Malaysian Voice Pathology Database. J. Otolaryngol. - Head and Neck Surgery.

[77] Mesallam T.A., Farahat M., Malki K.H., Alsulaiman M., Ali Z., Al-Nasheri A., Muhammad G. (2017). Development of the Arabic voice pathology database and its evaluation by using speech features and machine learning algorithms. J. Healthc. Eng..

[78] Bensoussan Y., Sigaras A., Rameau A., Elemento O., Powell M., Dorr D., Payne P., Ravitsky V., Bélisle-Pipon J.-C., Bahr R., Watts S., Bolser D., Siu J., Lerner-Ellis J., Rudzicz F., Boyer M., Abdel-Aty Y., Ahmed Syed T., Anibal J., Amraei D., Aradi S., Armosh K., Martinez A.S., Awan S., Bedrick S., Beltran H., Bernier A., Berrios M., Bevers I., Blatter A., Brito R., Brown A., Brown J., Cadillac L., Casalino S., Costello J., Dalal A., De Santiago I., Diaz-Ocampo E., Doherty-Kirby A., Ebraheem M., Eiseman E., Elmahdy M., English R., Evangelista E., Fletcher K., Gallois H., Garrett G., Gelbard A., Goldenberg A., Hanna K., Hersh W., Jain J., Jayachandran L., Jenney K., Jenkins K., Jo S., Johnson A., Kalia A., Kalia M., Khawa Z., Kostelnik C., Krause A., Krussel A., Lapadula E., Leo G., Levinsky J., Loewith C., Mahajan R., Maharaj V., Miao S., Michaels L., Mifsud M., Mikhael M., Moothedan E., Nafii Y., Neal T., Newberry K., Ng E., Nickel C., Peltier A., Pharr T., Pnacekova M., Pontell M., Premi-Bortolotto C., Rafatjou P., Rahman J.M., Ramos J., Rohde S., de Riesthal M., Rossi J., Russell L., Salvi Cruz S., Samuel J., Shah S., Shawkat A., Silberholz E., Stark J., Su L., Sudhakar S.G., Sutherland D., Swarna Mukhi V., Tang J., Taylor L., Toghranegar J., Tu J., Urbano M., Victor G., Vinson K., Wilke J., Wilson C., Zanin M., Zeng X., Zesiewicz T., Zhao R., Zisimopoulos P., Ghosh S. (2025). Bridge2AI-Voice: an ethically-sourced, diverse voice dataset linked to health information. PhysioNet.

[79] Naranjo L., Pérez C.J., Campos-Roca Y., Madruga M. (2021). Replication-based regularization approaches to diagnose Reinke’s edema by using voice recordings. Artif. Intell. Med..

[80] Kay Elemetrics Corp., Disordered Voice Database, version 1.03 (CD-ROM), (1994).

[81] Gómez-Vilda P., Fernández-Baillo R., Rodellar-Biarge V., Lluis V.N., Álvarez-Marquina A., Mazaira-Fernández L.M., Martínez-Olalla R., Godino-Llorente J.I. (2009). Glottal source biometrical signature for voice pathology detection. Speech. Commun..

[82] Wuyts F.L., De Bodt M.S., Molenberghs G., Remacle M., Heylen L., Millet B., Van Lierde K., Raes J., Van De Heyning P.H. (2000). The dysphonia severity Index: an objective measure of vocal quality based on a multiparameter approach. J. Speech, Lang. Hearing Research.

[83] Ghio A., Pouchoulin G., Teston B., Pinto S., Fredouille C., De Looze C., Robert D., Viallet F., Giovanni A. (2012). How to manage sound, physiological and clinical data of 2500 dysphonic and dysarthric speakers?. Speech. Commun..

[84] Fraj S., Schoentgen J., Grenez F. (2012). Development and perceptual assessment of a synthesizer of disordered voices. J. Acoust. Soc. Am..

[85] Natour Y.S., Saleem A.F. (2009). The performance of the time-frequency analysis software (TF32) in the acoustic analysis of the synthesized pathological voice. Journal of Voice.

[86] Dejonckere P., Schoentgen J., Giordano A., Fraj S., Bocchi L., Manfredi C. (2011). Validity of jitter measures in non-quasi-periodic voices. Part I: perceptual and computer performances in cycle pattern recognition. Logopedics Phoniatrics Vocology.

[87] Schiller I.S., Remacle A., Morsomme D. (2020). Imitating dysphonic voice: a suitable technique to create speech stimuli for spoken language processing tasks?. Logopedics Phoniatrics Vocology.

[88] Huang W.C., Halpern B.M., Violeta L.P., Scharenborg O., Toda T. (2022). Proceedings of the IEEE International Conference on Acoustics, Speech, and Signal Processing.

[89] Illa M., Halpern B.M., van Son R., Moro-Velazquez L., Scharenborg O. (2021). Proceedings of the 11th International Speech Communication Association Speech Synthesis Workshop.

[90] Liu G., Zhang T., Liu X., Hou X., Ding B., Fu D., Pang Z. (2024). PVR-vocoder: a pathological voice repair vocoder for voice disorders. IEEe J. Biomed. Health Inform..

[91] Yao D., Koivu A., Simonyan K. (2025). Applications of artificial intelligence in neurological voice disorders. World Journal of Otorhinolaryngology - Head and Neck Surgery.

[92] Peng X., Xu H., Liu J., Wang J., He C. (2023). Voice disorder classification using convolutional neural network based on deep transfer learning. Sci. Rep..

[93] Lakomkin E., Magg S., Weber C., Wermter S. (2018). Proceedings of the Conference on Empirical Methods in Natural Language Processing: System Demonstrations.

[94] Gonzalez J., Cervera T., Llau M.J. (2003). Acoustic analysis of pathological voices compressed with MPEG system. Journal of Voice.

[95] J. Mendes-Laureano, J.D. Arias-Londoño, J.I. Godino-Llorente, I. Cobeta-Marco, HUPA: a Castilian Spanish corpus of voice disorders, (2025). https://zenodo.org/records/17704572 (accessed March 19, 2026).

[96] He H., Garcia E.A. (2009). Learning from imbalanced data. IEEe Trans. Knowl. Data Eng..

[97] Buda M., Maki A., Mazurowski M.A. (2018). A systematic study of the class imbalance problem in convolutional neural networks. Neural Networks.

[98] Mehta D.D., Rudoy D., Wolfe P.J. (2012). Kalman-based autoregressive moving average modeling and inference for formant and antiformant tracking. J. Acoust. Soc. Am..

[99] Gómez-García J.A., Moro-Velázquez L., Arias-Londoño J.D., Godino-Llorente J.I. (2021). On the design of automatic voice condition analysis systems. Part III: review of acoustic modelling strategies. Biomed. Signal. Process. Control.

[100] Lyberg-Åhlander V., Rydell R., Fredlund P., Magnusson C., Wilén S. (2019). Prevalence of voice disorders in the general population, based on the Stockholm Public Health cohort. Journal of Voice.

[101] Patel R.R., Awan S.N., Barkmeier-Kraemer J., Courey M., Deliyski D., Eadie T., Paul D., Švec J.G., Hillman R. (2018). Recommended protocols for instrumental assessment of voice: american speech-language-hearing association expert panel to develop a protocol for instrumental assessment of vocal function. American Journal of Speech-Language Pathology.

[102] Awan S.N., Bahr R., Watts S., Boyer M., Budinsky R., Bensoussan Y. (2024). Validity of acoustic measures obtained using various recording methods including smartphones with and without headset microphones. J. Speech, Lang. Hearing Res..

[103] Nicastri M., Chiarella G., Gallo L.V., Catalano M., Cassandro E. (2004). Multidimensional Voice program (MDVP) and amplitude variation parameters in euphonic adult subjects. Normative study. Acta Otorhinolaryngologica Italica : Organo Ufficiale Della Società Italiana Di Otorinolaringologia e Chirurgia Cervico-Facciale.

[104] Kent R.D., Eichhorn J.T., Vorperian H.K. (2021). Acoustic parameters of voice in typically developing children ages 4–19 years. Int. J. Pediatr. Otorhinolaryngol..

[105] Nemr K., Simões-Zenari M., Cordeiro G.F., Tsuji D., Ogawa A.I., Ubrig M.T., Menezes M.H.M. (2012). GRBAS and cape-V scales: high reliability and consensus when applied at different times. Journal of Voice.

[106] Tsanas A., Little M.A., McSharry P.E., Ramig L.O. (2011). Nonlinear speech analysis algorithms mapped to a standard metric achieve clinically useful quantification of average Parkinson’s disease symptom severity. J. Royal Society Interface.

[107] Harar P., Galaz Z., Alonso-Hernandez J.B., Mekyska J., Burget R., Smekal Z. (2020). Towards robust voice pathology detection: investigation of supervised deep learning, gradient boosting, and anomaly detection approaches across four databases. Neural Comput. Appl..

[108] Pedregosa F., Varoquaux G., Gramfort A., Michel V., Thirion B., Grisel O., Blondel M., Prettenhofer P., Weiss R., Dubourg V., Vanderplas J., Passos A., Cournapeau D., Brucher M., Perrot M., Duchesnay É. (2011). Scikit-learn: machine learning in Python. J. Machine Learn. Res..

